# Expression and localization of apolipoprotein M in human colorectal tissues

**DOI:** 10.1186/1476-511X-9-102

**Published:** 2010-09-16

**Authors:** Guanghua Luo, Xiaoying Zhang, Qinfeng Mu, Lujun Chen, Lu Zheng, Jiang Wei, Maria Berggren-Söderlund, Peter Nilsson-Ehle, Ning Xu

**Affiliations:** 1 Comprehensive Laboratory, Third Affiliated Hospital, Suzhou University, Changzhou 213003, China; 2 Section of Clinical Chemistry & Pharmacology, Institute of Laboratory Medicine, Lunds University, S-221 85 Lund, Sweden

## Abstract

**Background:**

It has been well documented that apolipoprotein M (apoM) is principally expressed in the liver and kidney. However we found that there was weak apoM expression in other tissues or organs too, which could not be ignored. In the present study, we therefore examined apoM expression in human colorectal tissues including cancer tissues, cancer adjacent normal tissues, polyp tissues and normal mucosa as well as inflammatory mucosa.

**Methods:**

Tissue samples were collected from patients who underwent surgical resection or endoscopic examination. ApoM mRNA levels were determined by the real-time RT-PCR and apoM protein mass were examined by the immunohistochemistry.

**Results:**

ApoM protein can be detected in all colorectal tissues. However, apoM protein mass were significantly lower in the cancer tissues than its matched adjacent normal tissues, polyp tissues, normal mucosa and inflammatory mucosa. In parallel, apoM mRNA levels in the colorectal cancer tissues (0.0536 ± 0.0131) were also significantly lower than those in their adjacent normal tissues (0.1907 ± 0.0563) (*P *= 0.033). Interestingly, apoM mRNA levels in colorectal cancer tissues were statistic significant higher in the patients with lymph node metastasis than the patients without lymph node metastasis (*P *= 0.008). Patients under Dukes' C and D stages had much higher apoM mRNA levels than patients under Dukes' A and B stages (*P *= 0.034).

**Conclusion:**

It is concluded that apoM could also be expressed in human colorectal tissues besides liver and kidney. ApoM mRNA levels in the colorectal cancer tissues were significantly increased in the patients with lymph node metastasis. Whether increased apoM expression in the patients with lymph node metastasis being related to patients' prognosis and the physiopathological importance of apoM expression in colorectal tissues need further investigation.

## Introduction

Apolipoprotein M (apoM) was first identified and characterized in 1999 [1]. Human apoM gene is located in the major histocompatibility complex (MHC) class III locus on chromosome 6p21.31 (chromosome 17 in mouse) and contains six exons [1]. It has been well documented that human apoM is exclusively expressed in the hepatocytes in liver and tubular epithelial cell in kidney, and small amounts were also found in fetal liver and fetal kidney in most investigations [2]. However, in our previous study, during the human embryogenesis, relative high apoM mRNA could also be found in small intestine, stomach and skeletal muscle besides kidney and liver in the early stages of embryogenesis [3]. More recently, Calayir *et al*., reported that apoM could be expressed in human colorectal adenocarcinoma cell line, Caco-2 cells [4], which indicates that apoM might be expressed in human colorectal tissues too. In the present study we investigated whether apoM presented in human colorectal tissues and further investigated the difference of apoM expression pattern in colorectal tissues from patients with colorectal cancer and benign diseases.

## Materials and methods

### Patients and samples

Both colorectal cancer tissues and their adjacent normal tissues were collected from 20 patients (13 men and 7 women, aged from 38 to 82 years old, median age was 60 years old) who underwent surgical resection in the hospital. All patients' colon or rectal tumors were histologically classified as the adenocarcinoma and none patient had chemotherapy or radiotherapy before operation. Immediately after resection, small pieces of both tumor and its adjacent normal tissues were collected with snap-frozen and stored in liquid nitrogen before further experiments. Parts of samples were fixed in 10% (v/v) formalin and embedded in paraffin until use. Twenty three colorectal biopsy samples (7 normal mucosa, 6 inflammatory mucosa and 10 polyp tissues), during endoscopic examinations, were collected for the immunohistochemistry. The present study was approved by the local ethics committee and all patients gave informed consent.

### Total RNA extraction and real-time RT-PCR

Total RNA in tissues was extracted according to the manufacturer's instructions by using a total RNA purification kit (Biocolor BioScience and Technology Company, Shanghai, China). The quality of the RNA samples was determined by the absorbance measurements at 260/280 nm. Using the first strand cDNA synthetic kit (Fermantas, Vilnius, Lithuania) according to the manufacturer's instructions, 2 μg total RNA was reverse transcribed to cDNA. The mRNA levels of apoM and GAPDH were measured under real-time RT PCR by using TaqMan technology. The PCR primer sets were designed to amplify the apoM and GAPDH gene according to the information of GenBank, which are listed in Table 1. The real-time PCR reaction for each gene was performed in a 25 μL volume, in a glass capillary, containing 0.1 μL 100 mM each primer and probe, 2 μL cDNA, 2.5 μL 10 × buffer, 1.5 μL MgCl_2 _(25 mM), 0.5 μL dNTP (10 mmol/L), and Taq DNA polymerase 0.5 μL. Thermal cycling conditions included following steps: initial denaturation at 95°C for 2 min, followed by 40 cycles at 95°C for 5 sec and 60°C for 15 sec (apoM, 30 sec). All PCRs were performed on the LightCycler (Roche, Switzerland) real-time PCR system. The prospective amplicon of each gene was amplified and purified, then was ligated into the pMD19-T vector, and then ligated product was transformed into the E. Coli JM109 competent cells. In brief, a serial dilution of extracted plasmid DNA was used to generate a standard curve by plotting the cycle threshold versus the log initial copy number of input plasmid DNA. Both apoM and GAPDH standard curves achieved a very high-correlation coefficient (= 1.00). The ratio between the target gene and GAPDH was calculated to provide relative gene expression.

**Table 1:** Sequences of primers and probes

Gene (GenBank ID)	Primer/Probe	Sequence (5' to 3')
ApoM (AF118393.3)	Forward primer	tgccccggaaatggatcta
	Reverse primer	cagggcggccttcagtt
	Probe	FAM-cacctgactgaagggagcacagatctca-TAMRA
GAPDH (AF261085.1)	Forward primer	ggaaggtgaaggtcggagtc
	Reverse primer	cgttctcagccttgacggt
	Probe	FAM-tttggtcgtattgggcgcctg-TAMRA

### Immunohistochemistry

Normal mucosa, inflammatory mucosa, polyp tissues and both colorectal cancer tissues as well as their adjacent normal tissues from all patients were cut into sections under 3-μm thick for the immunohistochemical analyses. In brief, formalin-fixed, paraffin wax embedded tissues were dewaxed in xylene and rehydrated in graded ethanol solutions. Antigen retrieval was performed by incubating the slides at 100°C for 30 min in a 10 mmol/L citrate buffer (pH 6.0). Slides were cooled and immersed in a 0.3% hydrogen peroxide solution for 15 min to block endogenous peroxidase activity, and rinsed in PBS for 5 min, incubated with 5% bovine serum albumin at room temperature for 15 min to eliminate non-specific binding of primary antibody. Sections were then incubated with primary monoclonal antibodies against apoM (1:50 dilutions in PBS) (BD Biosciences) at 4°C overnight. PBS was used as negative control instead of the primary antibody. Then sections were incubated with HRP-labeled goat anti-mouse/rabbit secondary antibody (Ready to use, Maixin Biotechnology Limited Corporation, Fuzhou, China) for 30 min at room temperature. Diaminobenzidene (DAB) was used as the chromogen and hematoxylin as the nuclear counterstain. The sections were dehydrated, cleared and mounted.

### Evaluation of apoM immunohistochemical staining

Two pathologists were invited to examine the slides. In brief, five high-power fields (×200) were randomly selected. Intensity of apoM immunochemical staining and percentage of positive cells were assessed. The extent of the staining was categorized into five semi-quantitative classes based on the percentage of positive cells: 0 (< 5% positive cells), 1 (6-25% positive cells), 2 (26-50% positive cells), 3 (51-75% positive cells) and 4 (> 75% positive cells) [5]. The intensity of cytoplasmic and membrane staining was also semi-quantitatively determined on a scale of 0-3 as follow: 0 (negative), 1 (weak positive), 2 (moderate positive) and 3 (strong positive). Final staining score were obtained by multiplication of the intensity and the percentage scores and were represented as: 0 (negative), weak positive (1-4), moderate positive (5-8) and strong positive (9-12) [6]. In the present study we practically divided patients into two groups: low apoM expression group (score 0-8) and high apoM expression group (score 9-12).

### Statistical analysis

Results are expressed as means ± SE. Data were analyzed with the GraphPad Prism 4.0 software package (GraphPad Software, Inc., San Diego, California, USA). The paired *t *test, *χ*^2^-test and Mann Whitney test were applied for the statistical analyses. A *P *value less than 0.05 was considered to be significant.

## Results

### Immunohistochemical staining of apoM in normal mucosa, inflammatory mucosa, polyp tissues and cancer tissues

**Figure 1:**
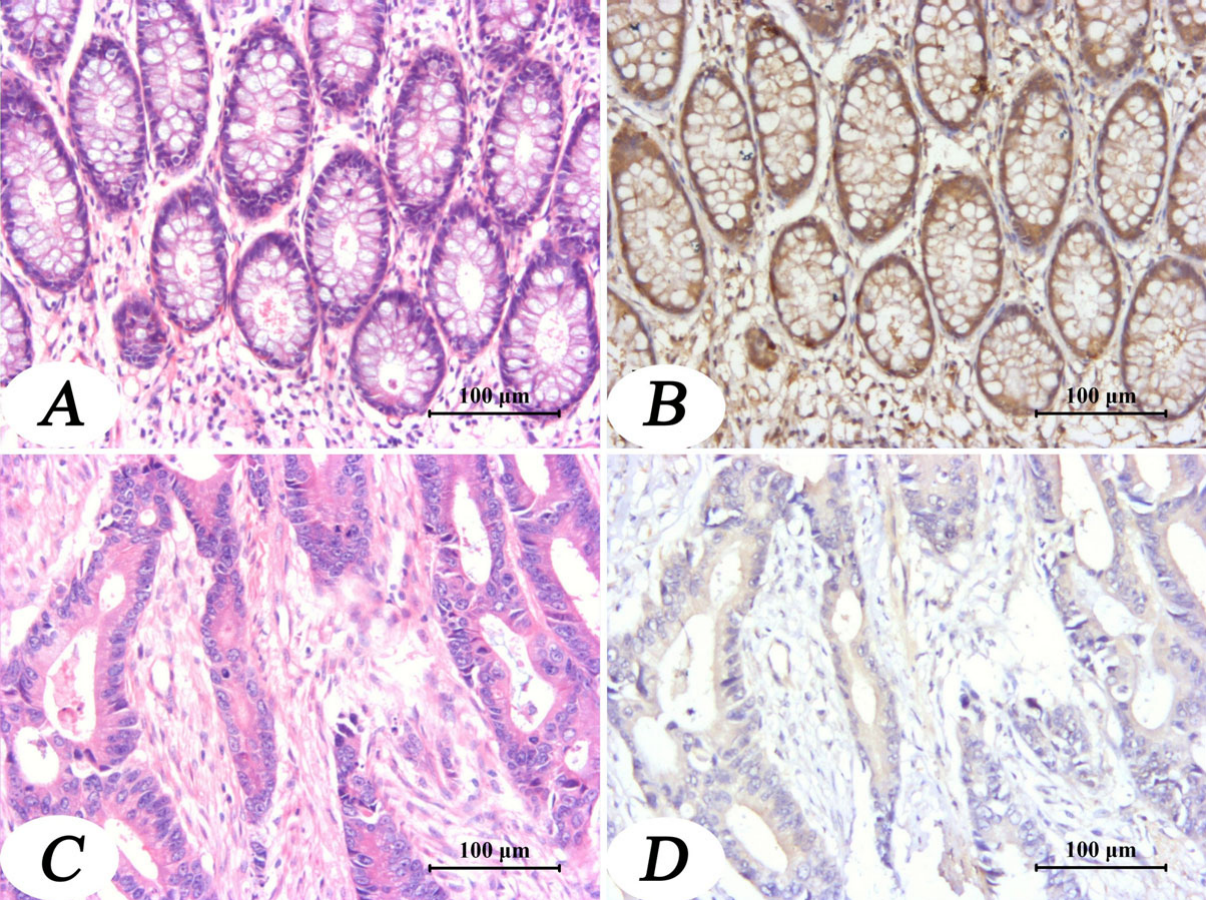
**Immunochemical staining of apoM in the colorectal tumor tissue and in its adjacent normal tissue**. Panels A and C show HE staining. Panels B and D show the immunohistochemical staining of apoM performed as described in material and methods. Brown color indicates positive staining. Magnification ×200.

**Figure 2:**
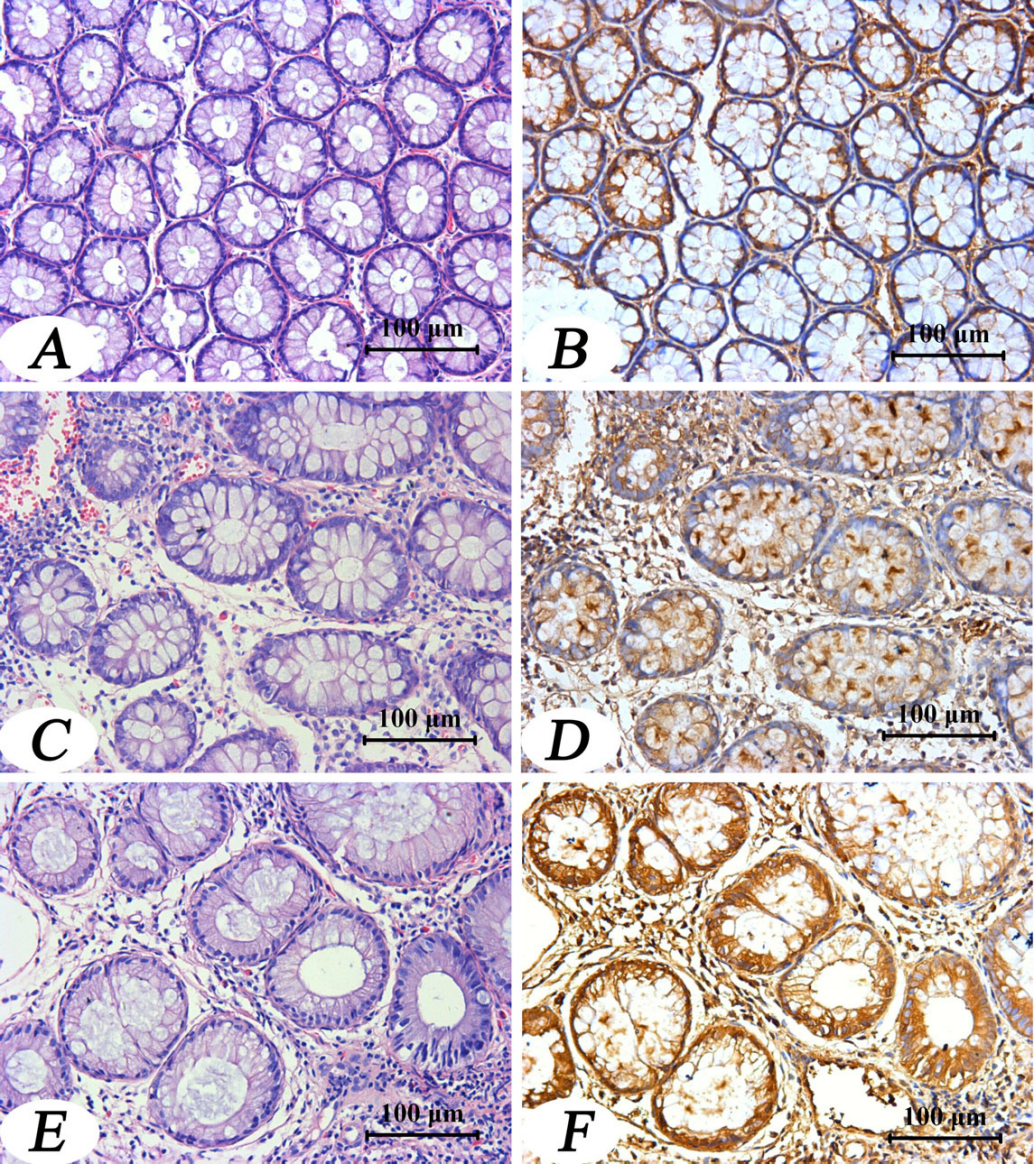
**Immunochemical staining of apoM in normal mucosa, inflammatory mucosa, and polyp tissue**. Panels A, C and E show HE staining of normal mucosa, inflammatory mucosa, and polyp tissue, respectively. Panels B, D and F show intensity of apoM immunostaining in the normal mucosa, inflammatory mucosa, and polyp tissue. Magnification ×200.

As shown in the figures 1 and 2, apoM protein could be detected in all colorectal tissues. However, apoM protein mass in the colorectal cancer tissues were significantly decreased compared to its adjacent tissues (Figure 1 and Table 2). And apoM protein mass was also much lower in the cancer tissues than those in the polyp tissues, normal mucosa and inflammatory mucosa (Table 3).

**Table 2:** Intensity of apoM immunochemical staining in the colorectal cancer tissues and their adjacent normal tissues

	Colorectal cancer tissue	Adjacent normal tissue	Odds ratio	*P*-value
**Low apoM HIS (score: 0-8)**	16 (80%)	3 (15%)		
			22.67	< 0.0001
**High apoM HIS (score: 9-12)**	4 (20%)	17 (85%)		

IHS: immunohistochemical staining

**Table 3:** Scores of apoM immunohistochemical staining in different colorectal tissues

Tissues	N	Median	Interquartile range	*P*-value*
Normal mucosa	7	10.50	3	**0.0109**
Inflammatory mucosa	6	9.75	4.5	**0.0356**
Polyp tissues	10	11	3.75	**0.0017**
Cancer tissues	20	5.5	4	

* The Mann Whitney test was applied, compared with cancer tissues.

### ApoM mRNA level and protein concentration in cancer tissue and its adjacent normal tissue

**Figure 3:**
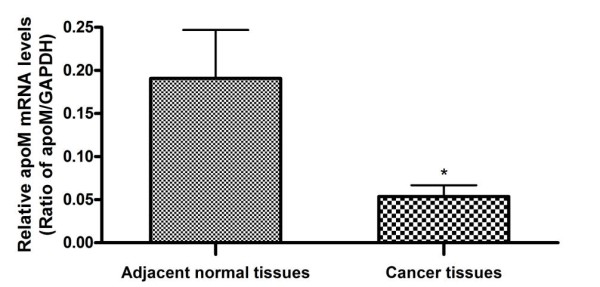
**ApoM mRNA levels in the colorectal tumor tissues and in their adjacent normal tissues**. The mRNA levels of apoM and GAPDH were determined by the real-time RT-PCR. Data are means ± SE (n = 20) and are analyzed with the paired student *t *test. *P < 0.05 vs. Adjacent normal tissues.

As shown in figure 3, apoM mRNA levels in colorectal cancer tissues (0.0536 ± 0.0131) were significantly lower than those in their adjacent normal tissues (0.1907 ± 0.0563) (*P *= 0.033). In parallel the apoM immunohistochemical staining were also much weaker in the cancer tissues than in their adjacent normal tissues (*P *< 0.0001, Table 2 and Figure 1). Under immunochemical staining, apoM protein was mainly located in normal columnar epithelial cells of the colorectal mucosa (Figure 1B) and non-regularly stained in the colorectal carcinoma cells (Figure 1D).

### Correlation of apoM mRNA levels and protein mass to patients' clinical characteristics

The present study clearly demonstrated that apoM mRNA levels in colorectal cancer tissues were significantly higher in the patients with lymph node metastasis than in patients without lymph node metastasis (*P *= 0.008), whereas apoM protein mass had no such difference between these two group patients (Table 4). Compared to patients under Dukes' A and B stages, patients under Dukes' C and D stages had higher apoM mRNA levels (*P *= 0.034), but not the apoM protein levels (Table 4). Neither apoM mRNA levels nor apoM protein concentrations in the colorectal cancer tissues were correlated to patients' sex, age, tumor stage, tumor size and tumor locations (Table 4).

**Table 4:** Correlation of apoM mRNA levels and protein levels with patients' clinical characteristics

Characteristic	n	ApoM mRNA levels		ApoM protein levels	
			
		Ratio of ApoM/GAPDH (Median)	*P*-value*	Staining score (Median)	*P*-value*
**Sex**			1.000		0.250
**Male**	13	0.0289		5	
**Female**	7	0.0386		7	
					
**Age**			0.362		0.648
**< 60**	9	0.0386		4	
**≥ 60**	11	0.0204		6	
					
**Tumor size (diameter)**			0.403		0.211
**< 6 cm**	11	0.0386		4	
**≥ 6 cm**	9	0.0204		6	
					
**Location**			0.481		0.529
**Colon**	10	0.0338		6.5	
**Rectum**	10	0.0271		4.5	
					
**Lymph node metastasis**			**0.008**		0.790
**without**	9	0.0151		5	
**with**	11	0.0514		6	
					
**Dukes' stage**			**0.034**		0.511
**A+B**	8	0.0172		5	
**C+D**	12	0.0450		6	
					
**Tumor (T) stage†**			0.337		0.694
**pT1+ pT2**	5	0.0193		6	
**pT3+ pT4**	15	0.0305		5	

* The Mann Whitney test was applied.

† The stage was determined by pathological (p) examination. T1: Tumor invading submucosa, T2: Tumor invading muscularis propria, T3: Tumor penetrating muscularis propria and invading subserosa, and T4: Tumor invading other organs or structures or perforating visceral peritoneum.

## Discussion

The tissue distribution, location of cellular expression, structure feature [1-3,7,8], and potential roles [9-15] as well as regulation of apoM [9,16-28] have been gradually elucidated since it was found and initially identified from human chylomicrons in 1999[1]. According to the literature, apoM is exclusively expressed in the hepatocytes in liver and in the tubular epithelial cells in kidney [2,3], and small amounts apoM expressed in the fetal liver and kidney too [3]. More recently, Calayir *et al*. reported that apoM expression could be up-regulated by the LXR agonist in human colorectal adenocarcinoma cell line, Caco-2 cells [4], which indicates that apoM might be also expressed in human colorectal tissues. However, our previous study [26] and Calayir's report demonstrated that LXR agonist could significantly inhibit apoM expression in HepG2 cells [4], which indicates that apoM in liver and in colon may have different physiological functions. We have previously reported that plasma apoM levels in the hepatocellular carcinoma (HCC) patients were significantly increased than those in the normal subjects [29]. However, both apoM mRNA levels and apoM protein mass in the HCC tissues were significantly lower than those in their adjacent tissues [30].

In the present study, we demonstrated that apoM was abundantly expressed in normal colorectal tissues and with inhibited levels found in the colorectal cancer tissues. Interestingly, apoM mRNA levels were significantly increased in the colorectal cancer tissues of patients with lymph node metastasis than the patients without lymph node metastasis. Moreover we demonstrated that apoM mRNA levels were much higher in the patients with Dukes' stages 3 and 4 than the Dukes' stages 1 and 2, which indicates that apoM mRNA levels in cancer tissues may have a potential positive correlation to the tumor progress, although apoM protein mass were not parallel increased in the patients with lymph node metastasis. This may suggest, although apoM expression is obviously decreased in the colorectal cancer tissues than in normal tissues, there are certain factors or mechanism could up-regulate apoM expression during the progress of the cancers. Perhaps apoM mRNA levels in colorectal cancer tissues can be determined for evaluating the metastasis, and further for patient's prognosis. Certainly it needs a standardized method for determining apoM mRNA level in the cancer tissues. In addition, it is still need to elucidate weather production of apoM in intestine influences plasma apoM pool in human.

Some proteins, such as alpha-fetoprotein (AFP), are normally produced in the developing embryo and fetus by hepatocytes, gastrointestinal cells and yolk sac cells. Synthesis of AFP stops at birth and its increased level is associated with pathological conditions [31]. In our previous study, apoM expression could also be found in small intestine, stomach and skeletal muscle in the early stages of embryogenesis [3]. We suspected whether apoM expression in adult colorectal tissues was influenced under cancer condition. In the present study, we examined apoM expression in normal mucosa, inflammatory mucosa, polyp and cancer tissues, which shows clearly that these tissues do express apoM, although apoM expression in the cancer tissues were significantly inhibited.

In conclusion, the present study revealed that apoM could also be abundantly expressed in human colorectal tissues, although the physiopathological importance of this expression and if it could influence plasma apoM pool are unknown yet. Moreover we demonstrated that apoM mRNA levels were significantly increased in the colorectal cancer tissues of patients with lymph node metastasis, although apoM expression in cancer tissues was generally much lower than in normal colorectal tissues. Determination of apoM mRNA in the surgical resected colorectal cancer tissues may have potential benefit for evaluating patient's prognosis.

## Competing interests

The authors declare that they have no competing interests.

## Authors' contributions

XZ and NX conceived the study. GL participated in the design of study, performed the statistical analysis and drafted the manuscript. QM, LC, LZ and JW carried out experiments. MBS and PNE provided substantive suggestions for revision and critically reviewed the manuscript. All authors have read and approved the final manuscript.
